# Multi-Features Fusion for Fault Diagnosis of Pedal Robot Using Time-Speed Signals

**DOI:** 10.3390/s19010163

**Published:** 2019-01-04

**Authors:** Yuhao Zhu, Zeyu Fu, Zhuang Fu, Xi Chen, Qi Wu

**Affiliations:** 1 State Key Laboratory of Mechanical System and Vibration, Shanghai Jiao Tong University, Shanghai 200240, China; yuhaozhu@sjtu.edu.cn (Y.Z.); summer0528@sjtu.edu.cn (Z.F.); 2 Shanghai Engineering Research Center of Civil Aircraft Health Monitoring, Shanghai Aircraft Customer Service Co., Ltd., Shanghai 200241, China; chenxi1@comac.cc; 3 School of Electronic, Information and Electrical Engineering, Shanghai Jiao Tong University, Shanghai 200240, China; wuqi7812@sjtu.edu.cn

**Keywords:** pedal robot, fault diagnosis, autoencoder, Treelet Transform, multi-features fusion

## Abstract

In order to realize automation of the pollutant emission tests of vehicles, a pedal robot is designed instead of a human-driven vehicle. Sometimes, the actual time-speed curve of the vehicle will deviate from the upper or lower limit of the worldwide light-duty test cycle (WLTC) target curve, which will cause a fault. In this paper, a new fault diagnosis method is proposed and applied to the pedal robot. Since principal component analysis (PCA), t-distributed stochastic neighbor embedding (t-SNE), and Autoencoder cannot extract feature information adequately when they are used alone, three types of feature components extracted by PCA, t-SNE, and Autoencoder are fused to form a nine-dimensional feature set. Then, the feature set is reduced into three-dimensional space via Treelet Transform. Finally, the fault samples are classified by Gaussian process classifier. Compared with the methods using only one algorithm to extract features, the proposed method has the minimum standard deviation, 0.0078, and almost the maximum accuracy, 98.17%. The accuracy of the proposed method is only 0.24% lower than that without Treelet Transform, but the processing time is 6.73% less than that without Treelet Transform. These indicate that the multi-features fusion model and Treelet Transform method is quite effective. Therefore, the proposed method is quite helpful for fault diagnosis of the pedal robot.

## 1. Introduction

**Figure 1:**
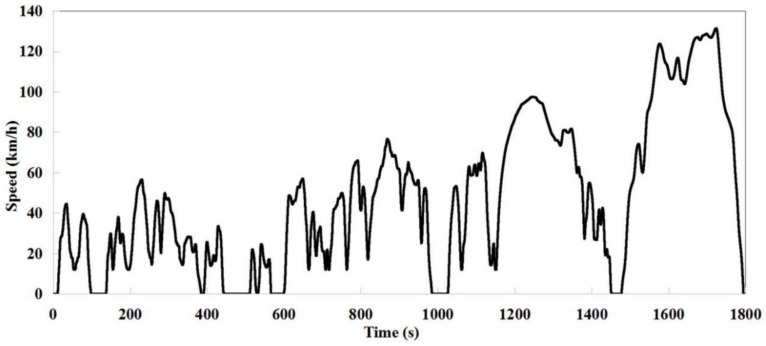
Curve of the worldwide light-duty test cycle (WLTC).

Before vehicle road tests or quality inspection, pollutant emission tests must be carried out. Over the past decade, these tests have been conducted by people who drive vehicles and follow the world light-duty test cycle (WLTC) target curve reported in Reference [1]. As shown in Figure 1, it is a time-speed curve that lasts 1800 s. In fact, when a vehicle is driven, the actual curve often deviates from the target curve. The noise in the test environment is too loud to be tolerated by operators, which may cause human error. In addition, research shows that although the deviation caused by robot control is less than that caused by human control, the number of deviations still needs to be reduced to almost zero. Generally, when the deviation between the actual curve and the target curve is greater than 2 km/h, a fault will occur. Different control parameters of pedal robot are needed for different types of faults. Therefore, how to identify fault types is still the most challenging part of robot control optimization.

Traditional fault diagnosis is analyzed by skilled engineers in time domain [2], which is difficult and time-consuming, especially in the case of large samples and/or high-dimensional samples. With the increase of data size, analysis becomes quite complex. It is necessary to reduce data size by feature extraction [3]. So far, many studies on feature extraction methods have been reported for the dynamic characteristics of fault signals [4]. One of the traditional feature extraction techniques is Fourier transform [5], but a Fourier coefficient represents a component that lasts for all time. Therefore, Fourier analysis is less suitable for nonstationary signals [6]. Other feature extraction algorithms, such as local mean decomposition, correlation coefficient, and kurtosis, can also extract features. In general, these features are directly used as input of the classifier. The calculation speed of these methods is fast, but they are not able to present adequate information of fault signals. Autoencoder, a deep learning algorithm, is used to extract more abstract features from the raw input. A major feature component is received via t-distributed stochastic neighbor embedding (t-SNE) algorithm or principal component analysis (PCA) method. These extracted feature components, or representations, are fused into a new matrix, which may contain more local major feature information. Thus, this work proposes a fusion methodology of multi-feature learning models via Autoencoder, t-SNE, and PCA.

PCA generates a set of orthogonal bases that capture the direction of the largest variance in training data [7]. Dunia et al. [8] used PCA for sensor fault identification via reconstruction. The disadvantage of PCA is that it can only extract linear features, while t-SNE can solve nonlinear problems. T-SNE, as proposed in Reference [9], is a very effective nonlinear dimension reduction technology, which was widely used recently. It is most commonly used to generate two-dimensional embedding of high-dimensional data in order to simplify cluster recognition [10]. However, PCA and t-SNE need to decompose the problem into different parts and merge the results in the final stage. In addition, most of the features in machine learning need to be recognized by experts, which is very troublesome. Deep learning has no such problems mentioned above, and can obtain the representations with sensitive and valuable feature through automatically learning from the original feature set [11]. In addition, because of the deep model [12], deep learning has stronger adaptability and stability. Therefore, an Autoencoder-based learning algorithm is used to extract abstract features from the raw input. The Autoencoder model also a special kind of neural network and is able to learn richer representation of input data, as well as represent the target output [13]. The aim of this model is to reconstruct input from the learnt representation [14]. Although the Autoencoder can automatically learn features, it also has some disadvantages. For example, when the training set is small, or the training sample does not follow the same distribution as the test sample, the accuracy of feature extraction will be reduced. Clearly, PCA, t-SNE, and Autoencoder have their own advantages and shortcomings over the feature learning. In order to make full use of their advantages, this work proposes a novel additive combination feature representation respectively from PCA, t-SNE, and Autoencoder. The features provided by them will contain more dimensional information of fault signals.

After feature extraction, the features are combined into a high-dimensional feature set. In order to speed up the calculation and visualize the dimension reduction results, it is necessary to reduce the dimension of feature set to three. Treelet Transform is a data dimension reduction technology [15]. It generates some sparse components, which is able to reveal the inherent structure of data [16]. After dimension reduction, a pattern classification method is needed. Gaussian process classifier based on Bayesian theory is one of the machine learning methods [17]. It can effectively classify nonlinear samples [18]. Gaussian process is used to learn the extracted features and present the fault detection results.

In this paper, a new fault diagnosis method based on multi-feature learning model is proposed. This method can identify fault type and help optimize the control of the pedal robot. The experimental results are compared with some typical feature extraction algorithms and the proposed model in terms of accuracy. The structure of this paper is as follows: Section 2 gives the proposed methodology, Section 3 gives experiment setup, Section 4 presents the results and discussion, and the conclusion is arranged in Section 5.

## 2. Methodology

The proposed fault diagnosis method for pedal robot mainly includes three steps: Feature extraction, dimension reduction, and fault classification. First, fault samples are extracted from the curve samples. Second, three-dimensional features are extracted from the fault samples by PCA, t-SNE, and Autoencoder, respectively. Next, since PCA, t-SNE, and Autoencoder cannot extract feature information adequately when they are used alone, and the types of features extracted by them are different, the features are combined into a nine-dimensional feature set to reduce information loss. Then, the feature set is reduced to three dimensions by Treelet Transform, thus, the calculation speed could be accelerated, and the fault samples could be visualized in three-dimensional space. Finally, after being classified by Gaussian process classifier, the accuracy and result of fault diagnosis are obtained.

### 2.1. Data Preprocessing

A fault sample contains two curves, namely, the target curve and the actual curve. Ten seconds before and after the midpoint of the deviation, the segment was extracted as a fault sample. Since the curve took a point at 0.1 s intervals, a fault sample had 200 points. In order to extract more features, data integration was necessary. The 200 points of the actual curve were combined with the 200 points of the corresponding target curve to form a 400-dimensional feature. In addition, this study also collected the time-displacement curves of the pedal robot’s two end-effectors. Therefore, the 400-dimensional feature and the 200 points of each end-effector displacement at the corresponding time were combined to form an 800-dimensional feature, which could be used as a fault sample. 

### 2.2. Feature Extraction

This work explores the feasibility of feature extraction by using PCA, t-SNE, and Autoencoder to extract three-dimensional features, respectively, then these three three-dimensional features are combined into a nine-dimensional feature matrix.

#### 2.2.1. Feature Extraction by PCA

The process of feature extraction by PCA consists of the following five stages:

Stage 1: Each sample is 800 dimensions, and 11430 samples constitute a sample array:

(1) $$x_{i}={\left(x_{i1},x_{i2},\ldots ,x_{ip}\right)}^{T},i=1,2,\ldots ,11430;p=1,2,\ldots ,800,$$

then, the sample elements are normalized as follows:

(2) $$Z_{ij}=\frac{x_{ij}-\bar{x_{j}}}{s_{j}},i=1,2,\ldots ,11430;j=1,2,\ldots ,800,$$

where $\bar{x_{j}}=\frac{\sum_{i=1}^{11430}x_{ij}}{11430},s_{j}{}^{2}=\frac{\sum_{i=1}^{11430}{\left(x_{ij}-\bar{x_{j}}\right)}^{2}}{11430-1},$ thus the normalized matrix **Z** is found.

Stage 2: Calculate the correlation coefficient matrix of the normalized matrix **Z**.

(3) $$R=\frac{Z^{T}Z}{11430-1},$$

Stage 3: Solve the eigenvalue of matrix **R**, then 800 eigenvalues are got to determine the principal components. The value of *m* is determined by the following equation [19]:

(4) $$\frac{\sum_{j=1}^{m}\lambda_{j}}{\sum_{j=1}^{800}\lambda_{j}}\geq 0.85,$$

For each $\lambda_{j},j=1,2,\ldots ,m$, solve the equations set $Rb=\lambda_{j}b$ to obtain the unit eigenvector $b_{j}^{0}$.

Stage 4: Convert the normalized indicator variables into principal components:

(5) $$U_{ij}=z_{i}^{T}b_{j}^{0},j=1,2,\ldots ,m,$$

where $U_{1}$ is the first principal component, $U_{2}$ is the second principal component and $U_{p}$ is the $p^{th}$ principal component. 

Stage 5: Comprehensive evaluation of m principal components.

In this paper, the total eigenvalues contribution of the first three eigenvalues was 99.37%. It indicates that the first three dimensions already contain enough information of the original sample. Thus, they were extracted as features of the high-dimensional fault samples.

#### 2.2.2. Feature Extraction by t-SNE

The d-dimensional input dataset is denoted by $\chi =\left\{x_{1},x_{2},\ldots ,x_{n}\right\}\subset C^{d}$. Before using t-SNE algorithm, PCA is used to initialize data. t-SNE computes a s-dimensional embedding of the points in χ, denoted by $y=\left\{y_{1},y_{2},\ldots ,y_{n}\right\}\subset C^{s}$, where *s*<*d*. The joint probability $p_{ij}$ measuring the similarity between $x_{i}$ and $x_{j}$ is computed as:

(6) $$p_{i|j}=\frac{exp\left(-∥x_{i}-x_{j}{∥}^{2}/2\sigma_{i}{}^{2}\right)}{\sum_{k\neq i}exp\left(-∥x_{i}-x_{k}{∥}^{2}/2\sigma_{i}{}^{2}\right)},\mathrm{and}\;p_{ij}=\frac{p_{i|j}+p_{j|i}}{2n}.$$

The bandwidth of the Gaussian kernel, $\sigma_{i}$, is often chosen such that the perplexity of $P_{i}$ matches a user defined value, where $P_{i}$ is the conditional distribution across all data point given $x_{i}$. The similarity between $y_{i}$ and $y_{j}$ in the low dimensional embedding is defined as:

(7) $$q_{ij}=\frac{{\left(1+∥y_{i}-y_{j}{∥}^{2}\right)}^{-1}}{\sum_{k\neq l}{\left(1+∥y_{k}-y_{l}{∥}^{2}\right)}^{-1}}.$$

t-SNE finds the points $\left\{y_{1},y_{2},\ldots ,y_{n}\right\}$ which minimize the Kullback-Leibler divergence between the joint distribution **P** of points in the input space and the joint distribution **Q** of points in the embedding space:

(8) $$C\left(y\right)=KL\left(P∥Q\right)=\sum_{i\neq j}p_{ij}log\frac{p_{ij}}{q_{ij}}.$$

The point y is initialized randomly, and the cost function C(y) is minimized using gradient descent. In this paper, *d* = 800, and *s* = 3. Thus, three-dimensional feature was extracted from the high-dimensional feature set.

#### 2.2.3. Feature Extraction by Autoencoder

An Autoencoder learns a feed-forward, hidden representation h(x) of the input x, and a reconstruction $\hat{x}$ could be got, which is as close as possible to x.

(9) h(x)=g(b+ωx),

(10) $$\hat{x}=sigm\left(c+\nu h\left(x\right)\right),$$

where ω and ν are matrices, *b* and *c* are vectors, g is a nonlinear activation function and sigm(a)=1/(1+exp(−a)). The loss function used in this paper is cross-entropy loss:

(11) $$l\left(x\right)=\sum_{d=1}^{D}=x_{d}log{\hat{x}}_{d}-\left(1-x_{d}\right)log\left(1-{\hat{x}}_{d}\right),$$

Training the Autoencoder corresponds to optimizing the parameters to reduce the average loss on the training examples. In this paper, the hidden layer h(x) has three neurons, which represent the features extracted from the input.

### 2.3. Dimension Reduction by Treelet Transform

After using PCA, t-SNE, and Autoencoder to extract three-dimensional features, respectively, the features are combined into a nine-dimensional feature set. Thus, a 11430×9 feature set matrix is extracted from the 11430×800 fault samples matrix. Then, this feature set is reduced to three-dimensional feature set by Treelet Transform.

Stage 1: Normalize the features extracted by PCA, t-SNE, and Autoencoder, respectively.

Stage 2: Define the clustering levels $l=0,1,2,\ldots ,L\left(L_{max}=p-1\right)$, where *p* is the dimension 9. At level l=0, each signal $x_{k}$ is represented by the original variables $x^{\left(0\right)}=\left[s_{0,1},s_{0,2},\ldots ,s_{0,p}\right]$ where $s_{0,k}=x_{k}$. Initialize similarity matrix and covariance matrix by the following Equation:

(12) $$\rho_{ij}=\frac{\Sigma_{ij}}{\sqrt{\Sigma_{ij}\Sigma_{ij}}},$$

with $\sum_{ij}=E{\left[\left(s_{i}-Es_{i}\right)\left(s_{j}-Es_{j}\right)\right]}^{T}$,

where $\rho_{ij}$ is the correlation coefficients of the similarity matrix $M_{\mathrm{ij}}$, and $M_{ij}=\left|\rho_{ij}\right|+\lambda \left|\sum_{ij}\right|$. Initialize the basis matrix $B_{0}=\left[\phi_{01},\phi_{02,}\ldots ,\phi_{0p}\right]$ as an identity matrix whose size is p×p. Initialize δ={1,2,…p}. For the *L*-level Treelet Transform, let l=1,2,…,L, repeat the following stages: 

Stage 3: At each level of the tree, the most similar variables are obtained according to the similarity matrix $M^{\left(l-1\right)}$, denote the two variables which have the maximum correlation coefficients as α and β:

(13) $$\left(\alpha ,\beta \right)=\arg\underset{i,j\in \delta }{\max}M_{ij}^{\left(l-1\right)},$$

Stage 4: Find the Jacobi rotation matrix:

(14) $$J\left(\alpha ,\beta ,\theta_{1}\right)=\left[\begin{array}{ccc} 1 & \cdots & 0\\ \vdots & \ddots & \vdots \\ 0 & \cdots & c \end{array}\right]\;J\left(\alpha ,\beta ,\theta_{1}\right)=\left[\begin{array}{ccccccc} 1 & \cdots & 0 & \cdots & 0 & \cdots & 0\\ \vdots & \ddots & \vdots & & \vdots & & \vdots \\ 0 & \cdots & c & \cdots & -s & \cdots & 0\\ \vdots & & \vdots & \ddots & \vdots & & \vdots \\ 0 & \cdots & s & \cdots & c & \cdots & 0\\ \vdots & & \vdots & & \vdots & \ddots & \vdots \\ 0 & \cdots & 0 & \cdots & 0 & \cdots & 1 \end{array}\right],$$

where $c=\cos\left(\theta_{l}\right)$ and $s=\sin\left(\theta_{l}\right)$, and the rotation angle $\theta_{1}$ can be obtained by:

(15) $$\left|\theta_{l}\right|\leq \frac{\pi }{4},\sum^{\left(l\right)}=J^{T}\sum^{\left(l-1\right)}J,\sum_{\alpha \beta }^{\left(l\right)}=\sum_{\beta \alpha }^{\left(l\right)}=0,$$

This transformation corresponds to a change of basis $B_{l}=B_{l-1}J$, and new coordinates $x^{\left(l\right)}=J^{T}x^{\left(l-1\right)}$. Update the similarity matrix $M^{\left(l\right)}=J^{T}M^{\left(l-1\right)}J$ accordingly.

Stage 5: Multi-resolution analysis. If $\overset{\left(l\right)}{\underset{\alpha \alpha }{\sum }}\geq \overset{\left(l\right)}{\underset{\beta }{\sum }}$, define the difference and sum as $d=x_{\beta }^{l}$ and $s_{1}=x_{\alpha }^{\left(l\right)}$. Define the scaling and detail functions $\phi_{1}$ and $\psi_{1}$ as columns α and β of the basis matrix $B_{l}$. 

Stage 6: Repeat stage 2 to stage 4 until reach the highest level l=L. Then, the final orthogonal basis matrix **B** can be obtained as follows:

(16) $$B={\left[\phi_{l},\psi_{1},\psi_{2},\ldots ,\psi_{l}\right]}^{T},$$

where $\phi_{1}$ is the scale of the highest level, and $\psi_{1}$ is the detail vector of the $1^{th}$ level. Then, the orthonormal treelet decomposition at level *l* can be obtained:

(17) $$x=\sum_{i=1}^{p-1}s_{l,i}\phi_{l,i}+\sum_{i=1}^{l}d_{i}\psi_{i},$$

Stage 7: Normalize the obtained eigenvectors after Treelet Transform. 

In this paper, the input was the 11430×9 feature set matrix, and the maximum level L=6. The output was three-dimensional normalized eigenvectors. The score of remaining three dimensions was 94.61%, which indicated that almost all the information had been extracted from the feature set matrix. So, the remaining three dimensions were input into the classifier as a representative of the high-dimensional feature set matrix.

### 2.4. Fault Classification by Gaussian Process Classifier

After feature extraction and dimension reduction, a 11430×3 matrix of fault samples is obtained. Then, these fault samples are trained and formed into four classes by Gaussian process classifier.

Assume the dataset is $D=\{\left(x_{i},y_{i}\right)|i=1,\ldots ,N\}$, where x is the input vector of dimension d and y is the class labels +1/−1. The input N×d matrix is denoted as X. Predictions for new inputs $x^{*}$ are made out of this given training data using the GP model. And GP binary classifier is done by first calculating the distribution over the latent function f corresponding to the test case,

(18) $$p\left(f_{*}|X,y,x^{*}\right)=\int p(f_{*}|X,x_{*},f)p(f|X,y)df,$$

where p(f|X,y)=p(y|f)p(f|X)/p(y|X) is the latent variable posterior. So, the probabilistic prediction is made by

(19) $${\bar{\pi }}_{*}=p\left(y_{*}=+1|X,y,x_{*}\right)=\int \sigma \left(f_{*}\right)p(f_{*}|X,y,x_{*})df_{*},$$

In this work, N=11430 and d=3. Since the outputs are divided into four classes, a multi-classification method is presented as: The first class consists of larger samples, and other three sample sets form the second class. This completes one classification and presents the classification probability of larger samples. Next, this step is repeated at the other three sample sets. Finally, one probability of every sample at each classification is obtained. The maximum probability is endowed to this sample. The accuracy is computed by comparing the predictive labels with the expert experience-based labels.

## 3. Experiment

**Figure 2:**
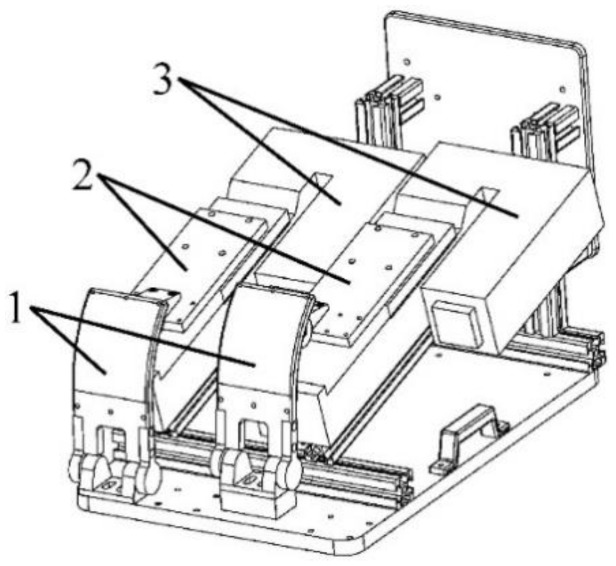
The axonometric drawing of pedal robot designed in this work. The numbers denote the following: 1—end-effectors of the robot, 2—sliders, and 3—servo motors.

**Figure 3:**
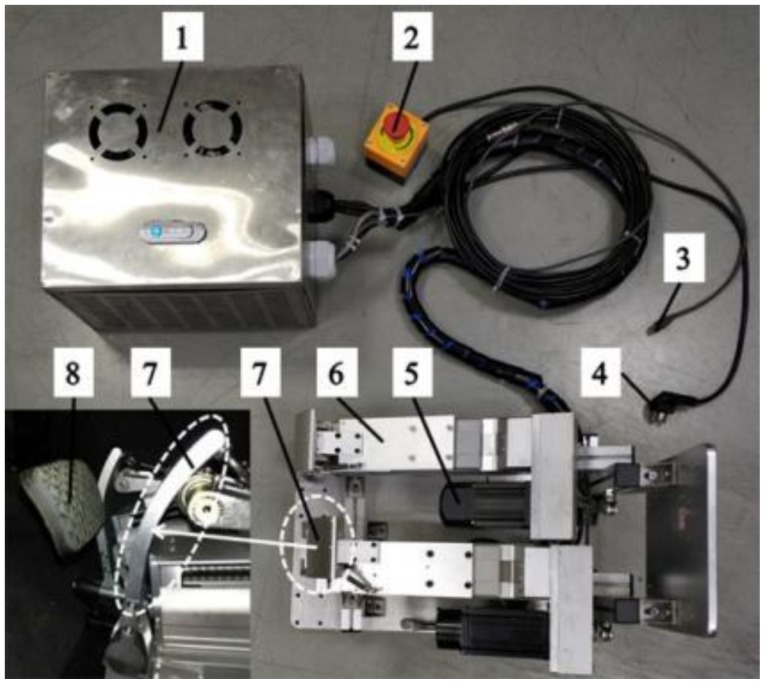
The robot system designed in this work. The numbers denote the following: 1—control box, 2—emergency stop button, 3—interface to the computer, 4—interface to the power supply, 5—servo motor, 6—slider, 7—end-effectors of the robot, and 8—brake pedal of the vehicle.

In order to realize the automation of pollutant emission tests without changing and disassembling the structure of vehicle products, a pedal robot instead of a human-driven vehicle is designed. As shown in Figure 2, the main components of the pedal robot are servo motors, sliders, and end-effectors. The servo motor pushes the end effector by controlling the slider. In order to improve the stability of control, the end-effector is designed to imitate the shape of human foot. As shown in Figure 3, the robot system consists of three parts: Control box, manipulator, and end effector.

**Figure 4:**
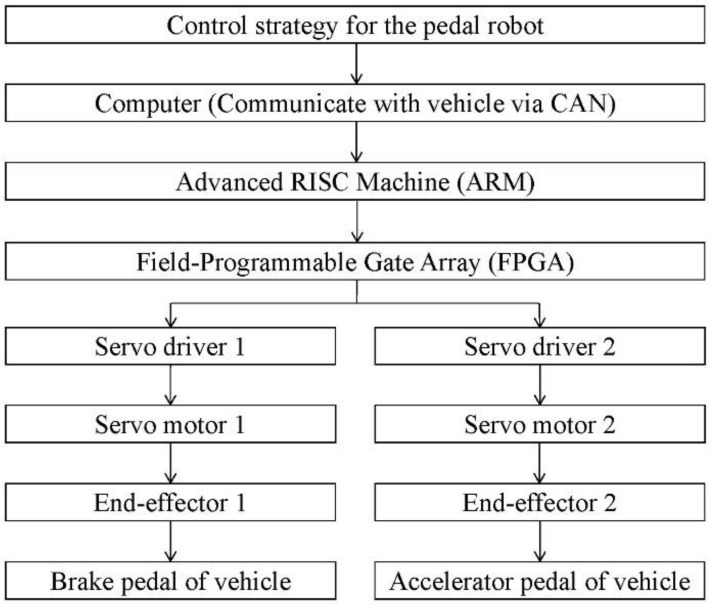
Procedures of robot control.

As shown in Figure 4, the operation process is as follows: First, the control strategy of the robot is transmitted to the advanced RISC machine (ARM) by computer. Next, ARM sends control signals to Field Programmable Gate Array (FPGA). Then, the servo motor is controlled by the servo driver. Finally, the servo motor pushes the end-effector to drive the brake pedal or acceleration pedal of the vehicle. During emission testing, the pedal robot will control the vehicle and make the time-speed curve of the vehicle follow the WLTC curve. The actual speed of the vehicle will be fed back to the computer every 0.1 s by Controller Area Network (CAN). Therefore, the faults can be analyzed according to the feedback information.

In this work, 50 different vehicles are used. After each vehicle test, the parameters of the robot control are adjusted according to the type and number of the faults, and the test is done again. On average, each vehicle undergoes about seven optimization processes, and finally, the number of faults in one test is reduced to almost zero. The fault appearance of each vehicle is not the same. By testing 50 vehicles and testing each vehicle several times, the usability of our method is improved, and the accuracy of fault prediction is also improved.

## 4. Results and Discussion

**Figure 5:**
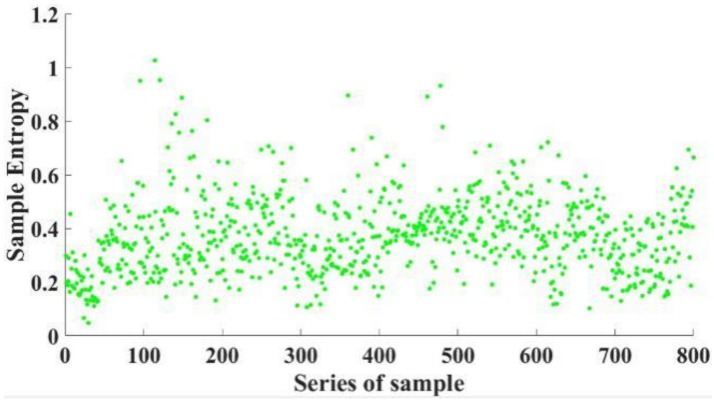
The sample entropy of 800 samples.

**Figure 6:**
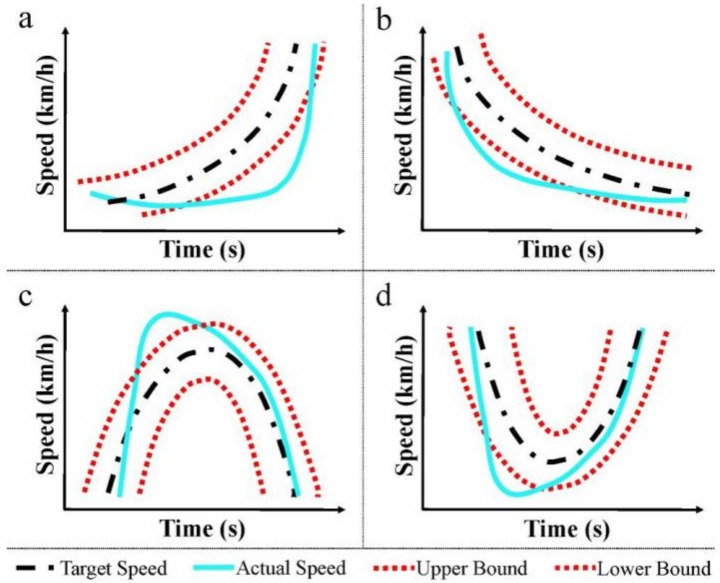
The schematic diagram of four types of faults: (**a**) Acceleration fault; (**b**) Deceleration fault; (**c**) Convex fault; and (**d**) Concave fault.

In this work, 346 pollutant emission tests are carried out, and 11,430 fault samples are obtained. Figure 5 shows the sample entropy of 800 samples. Based on expert experience, the fault samples are classified into four types, and each fault sample has a type label. The expert experience-based labels is the opinion of human operators. The operators are experienced, and by comparing the actual curve with the target curve, the type of fault can be correctly identified. Figure 6 shows the schematic diagram of four types of faults. According to the time-speed curve, faults can be classified into four types: Acceleration fault, deceleration fault, convex fault, and concave fault.

**Figure 7:**
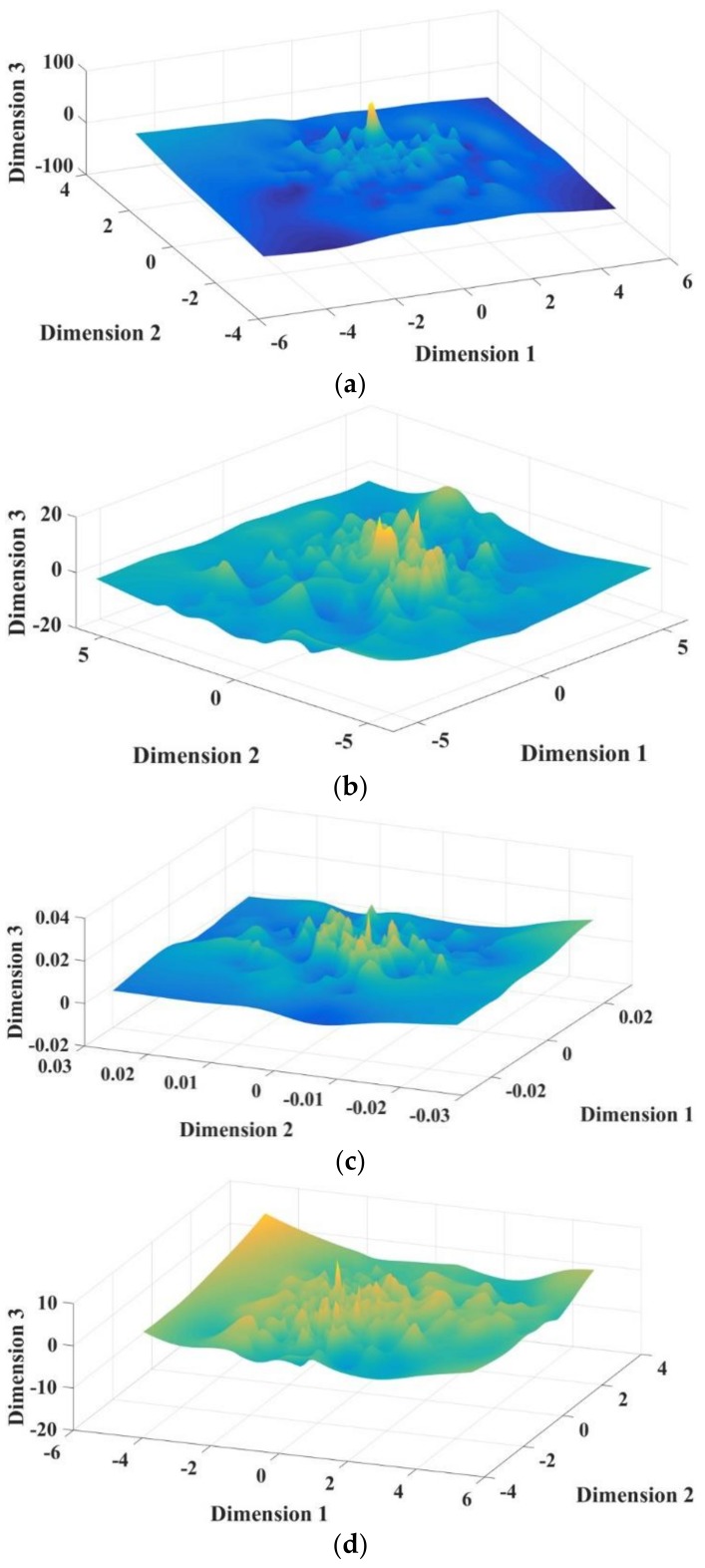
3D surface graph of three-dimensional features extracted by each algorithm. (**a**) Three-dimensional features extracted by principal component analysis (PCA); (**b**) Three-dimensional features extracted by t-distributed stochastic neighbor embedding (t-SNE); (**c**) Three-dimensional features extracted by Autoencoder; (**d**) Three-dimensional features extracted by Treelet Transform.

Gaussian process classification is quite time-consuming. If the dimension of the extracted feature is high, the total processing time will be long. But, if the dimension is low, the information will be lost. After many tests, it is found that the first three dimensions already contain enough information. It can not only reduce the dimension of data and accelerate the calculation speed, but also retain the feature information and the classification accuracy. Furthermore, 3D is good for visualization. After feature extraction, three-dimensional features are extracted from the fault samples by PCA, t-SNE, and Autoencoder, respectively. After dimension reduction by Treelet Transform, the feature set is reduced to a three-dimensional feature set matrix. Figure 7 is the 3D surface graph of three-dimensional features extracted by PCA, t-SNE, Autoencoder, and Treelet Transform, respectively. The values of x, y, and z coordinates are extracted three vectors.

The fault samples are classified into four types by Gaussian process classifier. Since ten-fold cross-validation has good performance [20], it is used in this paper. The average accuracy and standard deviation comparison between different methods is shown in Table 1. Method 4 is the method proposed in this paper, which has the minimum standard deviation and almost the maximum accuracy.

As shown in Table 1, when PCA, t-SNE, and Autoencoder are used to extract features as the input to Gaussian process classifier, respectively, the accuracy of Autoencoder is higher than that of t-SNE and PCA. This shows that the deep learning method is more effective than other two feature extraction methods. Since PCA, t-SNE, and Autoencoder cannot extract feature information adequately when they are used alone, and the feature types extracted by them are different, these features are combined into a nine-dimensional feature set as the input of Gaussian process classifier. And the accuracy of this method (Method 5) is 98.41%, which is much higher than that of PCA, t-SNE, and Autoencoder, respectively. This shows that the information loss can be effectively prevented by multi-features fusion.

In addition, as shown in Table 1, the processing time of Method 4 is far less than that of Method 5. Because the higher the dimension, the longer the time required for Gaussian process classification. The accuracy of Method 4 is only 0.24% lower than that of Method 5, but the processing time of one sample in Method 4 is 6.73% less than that of Method 5. These clearly prove that Treelet Transform can not only reduce the dimension of data and accelerate the calculation speed, but also retain the feature information and the classification accuracy. Therefore, the hybrid multi-models and Treelet Transform methodology is more effective than other methods.

**Figure 8:**
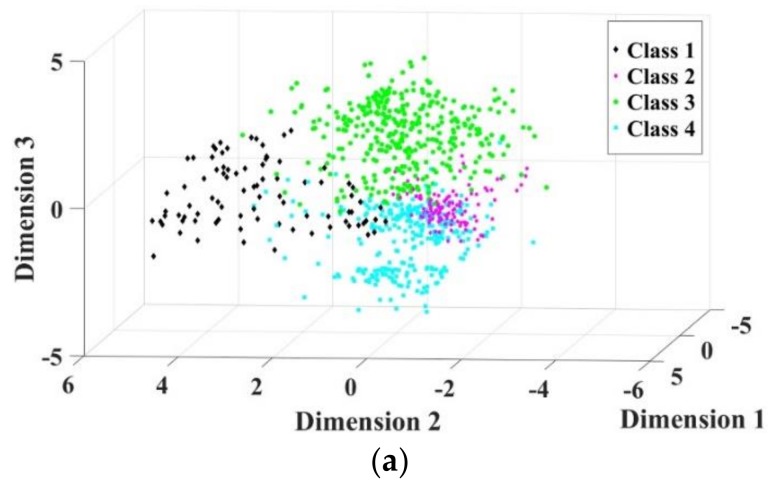

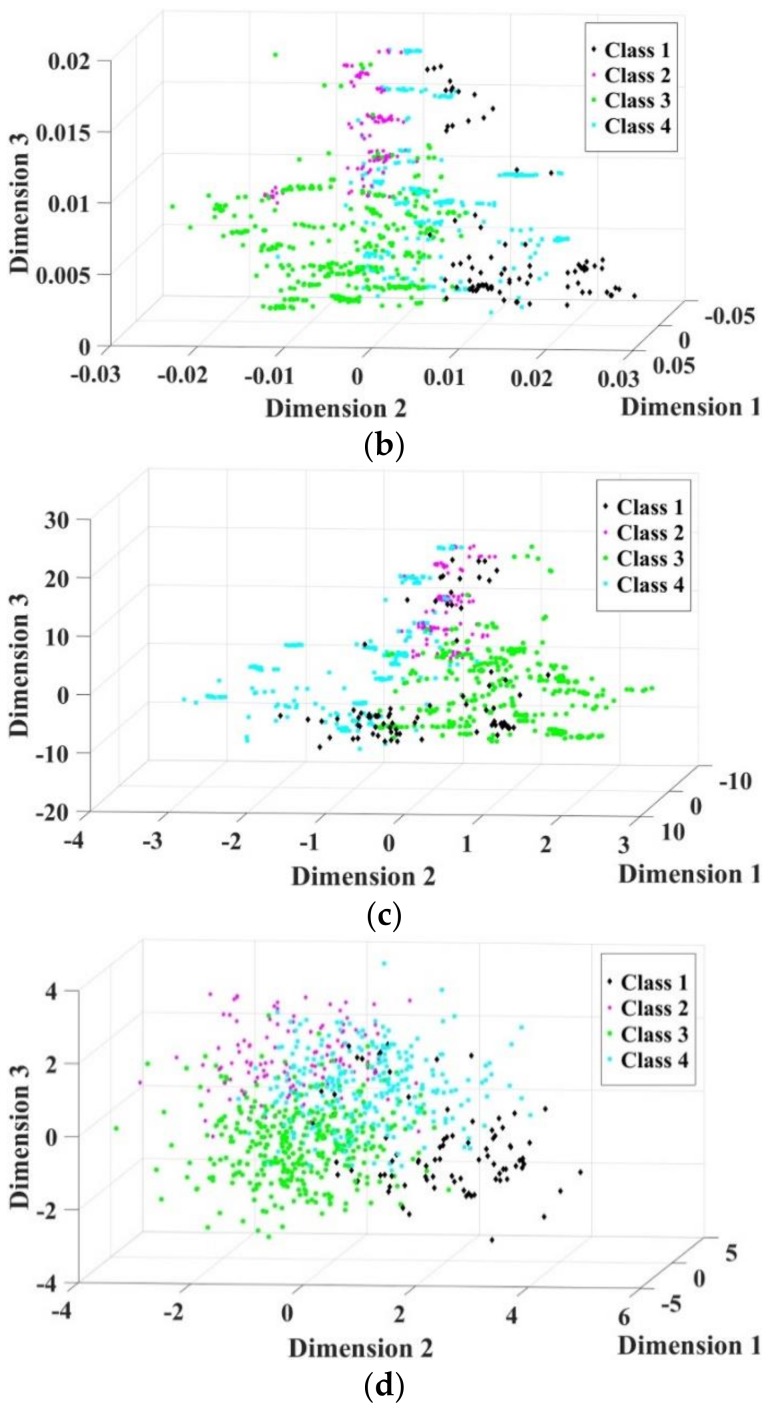
Distribution of 4 classes of faults. Class 1—convex fault; Class 2—concave fault; Class 3—acceleration fault; and Class 4—deceleration fault. (**a**) Distribution of Method 4; (**b**) Distribution of Method 3; (**c**) Distribution of Method 2; (**d**) Distribution of Method 1.

Furthermore, since the feature set is reduced to three dimensions, the fault samples could be visualized in three-dimensional space. This paper randomly selects some samples to make the figure clearer. As shown in Figure 8, the same position of three-dimensional axes are imposed in all the figures. The dots of Method 4 have more obvious interfaces than those of Method 1, Method 2, and Method 3. This also indicates that the method proposed in this paper produces great performance.

## 5. Conclusions

In order to realize the automation of pollutant emission tests, a pedal robot is designed instead of a human-driven vehicle. The actual time-speed curve of the vehicle should follow the WLTC target curve. However, sometimes the actual curve will deviate from the upper or lower limit of the target curve, which will cause a fault. In this paper, a new fault diagnosis method is proposed and applied to the fault diagnosis of pedal robot. There were 346 pollutant emission tests carried out, and 11430 fault samples obtained for fault diagnosis. To find more effective feature information from the fault samples, a deep learning algorithm, Autoencoder, is used for feature extraction. The results show that it is better than other feature extraction algorithms. Since PCA, t-SNE, and Autoencoder cannot extract feature information adequately when they are used alone, and the feature types they extract are different, three types of feature components extracted by PCA, t-SNE, and Autoencoder are fused to form a nine-dimensional feature set. Then, the feature set is reduced into three-dimensional space via Treelet Transform. Finally, the fault samples are classified into four types by using Gaussian process classifier. The experimental results show that the proposed method has the minimum standard deviation, 0.0078, and almost the maximum accuracy, 98.17%. Compared with the method using only one algorithm to extract features, the proposed method has much higher classification accuracy. This shows that the information loss can be effectively prevented by multi-features fusion.

Moreover, Treelet Transform is used to reduce dimension of feature set, so that the fault samples can be visualized in three-dimensional space. The accuracy of the proposed method is only 0.24% lower than that without Treelet Transform, but the processing time of one sample is 6.73% less than that without Treelet Transform. These clearly prove that Treelet Transform can not only reduce the dimension of data and accelerate the calculation speed, but also retain the feature information and the classification accuracy. It indicates that the hybrid multi-models and Treelet Transform method is quite effective. Therefore, the proposed method can efficiently identify the type of the faults, which can help optimize the control of the pedal robot in pollutant emission test.

## Figures and Tables

**Table 1:** Comparison among different methods.

Method	Feature Extraction Algorithm	Dimension Reduction Algorithm	Accuracy	Standard Deviation	Processing Time of One Sample
1	PCA	None	87.60%	0.0197	9 ms
2	t-SNE	None	91.53%	0.0112	72 ms
3	Autoencoder	None	93.28%	0.0136	23 ms
4	PCA + t-SNE + Autoencoder	Treelet Transform	98.17%	0.0078	97 ms
5	PCA + t-SNE + Autoencoder	None	98.41%	0.0094	104 ms
